# Evaluation of the Effect of Axial Wall Modification and Coping Design on the Retention of Cement-retained Implant-supported Crowns

**DOI:** 10.15171/joddd.2015.007

**Published:** 2015-03-04

**Authors:** Reza Derafshi, Ahmad Hasan Ahangari, Kianoosh Torabi, Mitra Farzin

**Affiliations:** ^1^Assistant Professor, Department of Prosthodontics, Faculty of Dentistry, Shiraz University of Medical Sciences, Shiraz, Iran; ^2^Associate Professor, Department of Prosthodontics, Faculty of Dentistry, Shiraz University of Medical Sciences, Shiraz, Iran; ^3^Biomaterial Research Center, Faculty of Dentistry, Shiraz University of Medical Sciences, Shiraz, Iran

**Keywords:** Dental abutments, dental implants, dental implant abutment design, implant supported, retention

## Abstract

***Background and aims.*** Because of compromised angulations of implants, the abutments are sometimes prepared. The purpose of this study was to investigate the effect of removing one wall of the implant abutment on the retention of cement-retained crowns.

***Materials and methods.*** Four prefabricated abutments were attached to analogues and embedded in acrylic resin blocks. The first abutment was left intact. Axial walls were partially removed from the remaining abutments to produce abutments with three walls. The screw access channel for the first and second abutments were completely filled with composite resin. For the third and fourth abutments, only partial filling was done. Wax-up models were made by CAD/CAM. Ten cast copings were fabricated for each abutment. The copings of fourth abutment had an extension into the screw access channel. Copings were cemented with Temp Bond. The castings were removed from the abutment using an Instron machine, and the peak removal force was recorded. A one-way ANOVA was used to test for a significant difference followed by the pairwise comparisons.

***Results.*** The abutments with opened screw access channel had a significantly higher retention than the two other abutments. The abutment with removed wall and no engagement into the hole by the castings exhibited the highest retention.

***Conclusion.*** Preserving the opening of screw access channel significantly increases the retention where one of the axial walls of implant abutments for cement-retained restorations is removed during preparation.

## Introduction

Retention, in prosthodontics, is defined as the inherent ability to overcome the dislodging forces which are present along the path of placement.^1-2^ Factors influencing the retention of a cemented crown can be classified into three categories: the tooth preparation technique (degree of convergence, surface area and texture, height of the preparation), the casting (texture of interior surface, relative adaptation of the crown to the preparation, influence of pre-planned openings in the casting), and the cements (the type and the viscosity).^2^

Implication of dental implants in the rehabilitation of partially edentulous patients has become a well–recognized, accepted clinical method with predictable long-term success.^3^ There are two different techniques of retaining fixed implant supported restorations: Screw retention and cementation.^3-6^ The main advantage of screw-retained prosthesis is retrievability of the supra-structures.^3,5-7^, Screw retention is often preferred in presence of limited interocclusal space, long cantilevers, and deep submucosal placement of implant shoulder.^5^ As techniques continue to evolve, the survival rates of implant-retained restorations are improving. Therefore, the use of cement-retained implant-supported restorations has increased.^7^

The advantages of cement-retained, implant supported restorations are their ability to optimize occlusal interdigitation,^7-9^ enhancement of the esthetics in areas that would otherwise be the locations of screw access holes,^5-9^ providing a passive fit,^5-9^—which may actually improve loading characteristics,^5,7,9^ and a reduction of complications such as elimination of occlusal screw loosening,^5,8^ as well as fracture of porcelain, cost, and time.^5^ Many of the factors influencing the retention of cement-retained, implant-supported restorations can be inferred from previous studies on natural abutments. The majority of abutment preparation designs and cementation techniques now imitate conventional fixed prosthodontic procedures for natural teeth.^2-10^

Ideally, the cement on an implant-retained restoration would provide sufficient retention to prevent it from loosening and allow the restoration to be removed without damaging the abutment, the restoration or the peri-implant tissues.^11^ It has been recommended that the ideal tapering and the longer walls of implant abutments support the use of provisional cements. There is not enough evidence regarding the most suitable type of cement and the behavior of provisional cements over time.^12^

The presence of a screw access channel and having varying number of axial walls are the main geometrical difference between an implant abutment and a natural tooth preparation. Despite the stated differences, there are only a limited number of publications evaluating the effect of modified screw access channel on the retention of cemented restorations.^2^ Tan et al^2^demonstrated that the number and the position of the axial walls of implant abutments can be designed or amended to improve the retentive strength of cemented crowns which is unlike natural abutments. Emms et al^10^ also state that the method employed to fill the screw access channel of implant abutments can have an influence on the retention of coronal restorations cemented with Temp Bond. Naik et al^7^ confirmed that engagement of a casting cemented with Temp Bond into the screw access channel of an implant abutment considerably increases the retention. Therefore, a study deemed necessary to evaluate if there were any relationships between axial wall modification or coping design and the retention of the cement-retained implant-supported crowns.

The purpose of the present study was to investigate the effect of three different methods on the retention of cement-retained crowns when one wall of the implant abutment is removed. The null hypothesis was: there would be no significant difference in the retention of cemented crowns whether the implant abutments are intact or have lost one wall with engaging the screw access channel or without it.

## Materials and Methods

Four prefabricated straight abutments (SM; DIO, Busan, Korea) were attached onto four corresponding implant analogues. Each abutment was tightened with digital pressure with a hex driver. The interface diameter for all implant abutments was 4.1 mm. The height of the selected abutments was 5.5 mm. The abutment-analogue complexes were vertically mounted in individual acrylic resin blocks (AcroPars 200; Marlic Medical Industries Co., Tehran, Iran) with a dental surveyor (Ney Dental- Intl, Bloomfield, USA). The acrylic resin was left 1 mm lower than the implant-abutment joint.

The first abutment was left intact without any modification as control group. The three other abutments were prepared by using a tapered carbide bur to remove 4 mm of the height of the flat walls of them.

**Figure 1.**
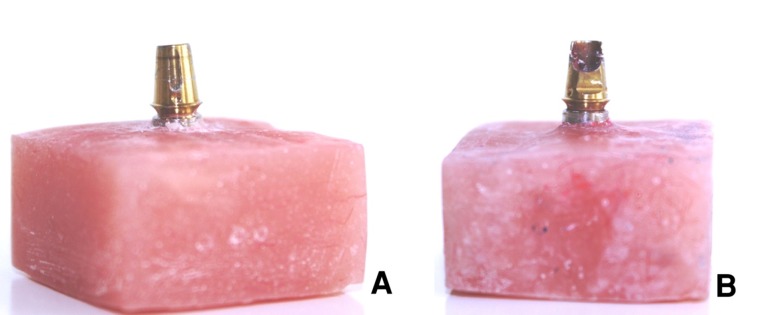
The intact abutment was used for fabricating 30 castings (A). The abutment with opened screw access channel was used for making 10 castings with extensions into the channel (B).
- For the second abutment, a cotton pellet was placed on top of the abutment screw and the screw access channel was filled with the same composite resin to restore the original contour of the abutment.
- For the third abutment, a cotton pellet was placed on top of the abutment screw and the opening of the screw access channel was preserved.
- For the fourth abutment, a cotton pellet was placed on top of the abutment screw and the screw access channel was filled with the same composite resin up to the top of the remaining wall so that the 4 mm opened window was preserved (Figure 1B, Table 1).

- For the first abutment, a cotton pellet was placed on top of the abutment screw and the screw access channel was completely filled with composite resin (chemical cure; PRIME-DENT, Miami, USA; Figure 1A).

**Table 1:** Description of the groups of abutments tested in the study

Abutment	Modification	Screw access channel
1	Intact	Filled
2	One wall removed	Filled to restore the original con- tour
3	One wall removed	Open without engagement the castings into it
4	One wall removed	Open with engagement the cast- ings into it

**Figure 2.**
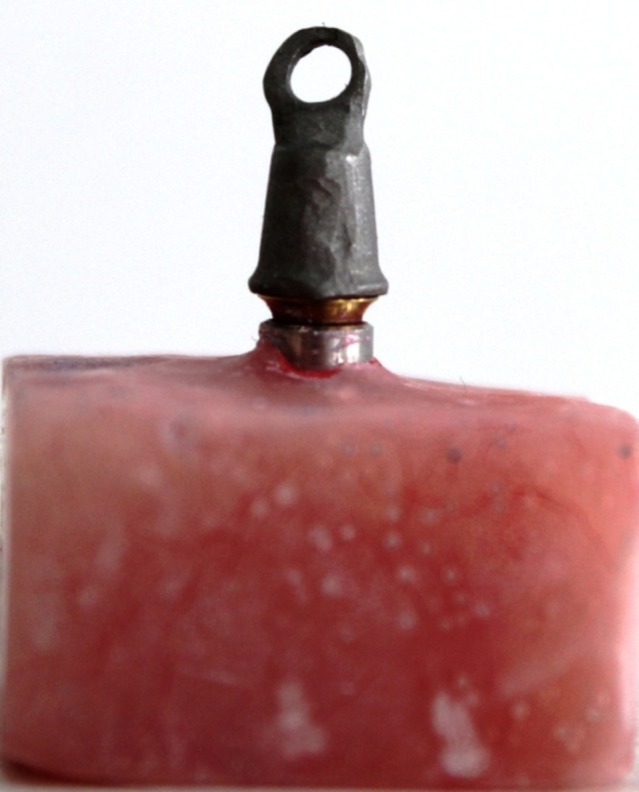
Scanned image of the intact abutment (A). Scanned image of the abutment with opened screw access channel (B).

**Figure 3.**
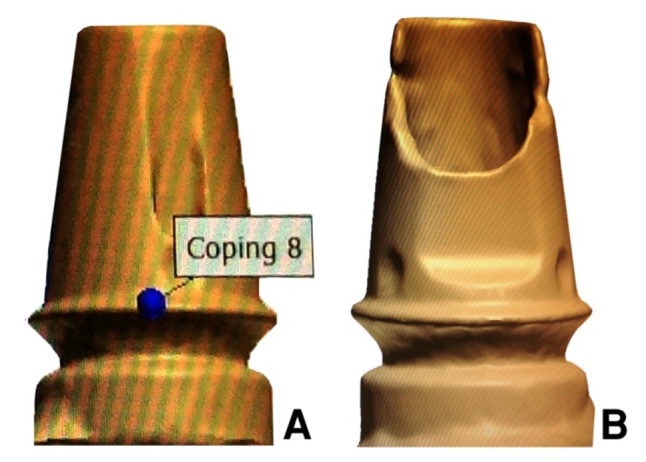
The casting with a loop attachment.

Thirty wax copings were made directly on the first abutment by CAD/CAM technique using 3 Shape D810 Scanner (3 Shape, Copenhagen, Denmark) and CAD/CAM machine (imes-icore, GmbH, Germany) to make the wax copings (Laserdenta-CAD WAXGOLD, Bergheim, Germany; Figure 2). 20 μm space was defined between each wax coping and the abutment to within 2 mm of the margin. A loop attachment was added on the occlusal surface of each wax coping before casting. Wax copings were sprued, invested with phosphate-bonded investment material (ERNST HINRICHS, GmbH, Germany), and cast with base metal alloy (4all; Ivoclar Vivadent, Liechtenstein; Figure 3).

Ten wax copings were made on the fourth abutment by the same CAD/CAM technique, except that 40 μm space was defined between each wax coping and the screw access channel walls and wax was introduced into the channel and corresponding castings were constructed as ten other castings. If any surface irregularity was present, it was removed with a bur.

The first thirty castings were divided randomly into three groups. Each group contained ten castings and were related to one of the first, second and third abutments, and the remaining ten castings with extension into the screw access channel were related to the fourth abutment.

Weighed amounts of Temp Bond (Kerr Italia srl, Scafati, Italy) were used for the cementation of the castings on the implant abutments and mixed for 30 seconds in proportions according to the manufacturer’s instructions. The mixed cement was placed in the castings and the castings were seated onto the abutments with finger pressure, and placed under a 5-kg pressure for 5 minutes. Excess cement was removed with a plastic scaler. The assemblies were then stored in 100% humidity at 37ºC for 24 hours.

The castings were attached to a universal testing machine (Zwick CombH- &Co/Roell.Z020, Ulm, Germany) by clamping them directly onto the loop attachment. The machine was used to apply vertical tensile forces at a crosshead speed of 5 mm per minute, to dislodge the castings from the abutments. The peak load to dislodgement was documented (N) and used to indicate the retentive values. Before testing each new casting, abutments were cleaned of remaining cements. To compare the groups, one-way analysis of variance (one-way ANOVA) and pairwise comparisons test (LSD) were used.

## Results

The results of one-way ANOVA revealed a significant difference between the groups (P = 0.019). To achieve the homogeneity of variances in one-way ANOVA, the data of peak loads (retention) was changed to natural logarithm (Ln; Table 2).

**Table 2:** Mean ± SD of retention values and the results of one-way ANOVA and pairwise comparisons (LSD) tests of the studied groups

Group	Retention (N)	Ln (retention)^*^	P(one-way ANOVA)	Significant pairwise comparisons (LSD P value)
1	46.88 ± 10.16	3.82 ± 0.24		3 vs 1 (0.016)
2	46.31 ± 9.06	3.82 ± 0.20	0.019	3 vs 2 (0.015)
3	65.30 ± 18.85	4.13 ± 0.33		4 vs 1 (0.038)
4	62.25 ± 17.92	4.09 ± 0.31		4 vs 2 (0.034)
^*^Retention was changed to natural logarithm (Ln) to obtain homogeneity of variances in one-way ANOVA.				

Pairwise comparisons test (LSD) revealed that the abutments of the third and fourth groups in which one wall was removed and the screw access channel openings were preserved, had a significantly higher mean peak load to dislodgement than two other groups that their screw access channels were filled with composite (Table 2).

There was no significant difference between the first and second groups and between the third and fourth groups. The abutment of the third group in which one wall of the abutment was prepared and there was no extension of the castings in to the screw access channel exhibited the highest retention. The lowest mean retention value was seen in the second group that after removing one wall of the abutment, the screw access channel was restored to the original contour with composite (Table 2).

## Discussion

The results of this study support rejection of the null hypothesis that there would be no significant difference in the retention of cemented crowns whether the implant abutments are intact or have lost one wall with engaging the screw access channel or without it. Removing one wall of the abutment and leaving the screw access channel opened, either with engaging or without engaging the screw access channel, had significant influences on the retention. The higher retention of these abutments may be contributed to increasing the surface area due to the penetration of the cement into the screw access channel. The presence of internal axial walls compensated for the decrease in retentive surface area as a result of removing one wall. These findings are in line with the general consensus that retention is positively correlated with the surface area of the abutment—whether a natural tooth or an implant.^2,10,13^ Another cause would be the surface roughness of the internal walls, as according to Tan et al,^2^ unlike the external axial walls, the internal walls are not coated with the smooth titanium nitride. This may have contributed to the observed results because the roughness of the surface preparation can have a pronounced effect on the retention.^2,14,15^

Another possible explanation for the improved retention seen in the abutments with one removed wall compared to abutments with 4 walls was the presence of an open screw access channel that may act as an internal vent, allowing for more complete seating of the castings, as explained previously in the literature.^2^ This venting effect may explain the higher retention of the abutments with opened screw access channel and no engaging of the channel in comparison to abutments with opened screw access channel and engaged channel. Contrary to this finding, however, Naik et al^7^ concluded that engaging the screw access channel of an implant abutment can significantly improve retention.

This study also confirmed the explanation of Emms et al^10^ that complete filling of the screw access channel before cementation could reduce the removal force of a coronal restoration cemented with Temp Bond. The results were very similar between the intact abutment group and the group in which the screw access channel was filled with composite resin. Clinically, this finding means that if one wall of the abutment is removed, filling the hole with composite to the original contour produces the same retention as the intact abutment.

The limitations of this study should be noted, since it only investigated retention and not resistance. Clinically, the removal of prosthodontic castings might not apply forces along a single withdrawal path.

## Conclusion

Within the limitations of this *in vitro* study, when one of the axial walls of implant abutments for cement-retained restorations is removed during preparation, preserving the opening of screw access channel significantly increases retention. This finding is consistent regardless of engaging the opening with casting into the abutment screw access channel or not.
